# *Bartonella* Endocarditis in Spain: Case Reports of 21 Cases

**DOI:** 10.3390/pathogens11050561

**Published:** 2022-05-10

**Authors:** Lara García-Álvarez, Concepción García-García, Patricia Muñoz, María del Carmen Fariñas-Álvarez, Manuel Gutiérrez Cuadra, Nuria Fernández-Hidalgo, Elisa García-Vázquez, Encarnación Moral-Escudero, María del Mar Alonso-Socas, Dácil García-Rosado, Carmen Hidalgo-Tenorio, Fernando Domínguez, Josune Goikoetxea-Agirre, Juan Carlos Gainzarain, María Ángeles Rodríguez-Esteban, Xerach Bosch-Guerra, José A. Oteo, on behalf of Grupo de Apoyo al Manejo de la Endocarditis infecciosa en España (GAMES)

**Affiliations:** 1 Infectious Diseases Department, San Pedro University Hospital-Center for Biomedical Research from La Rioja (CIBIR), 26006 Logroño, Spain; lgalvarez.ext@riojasalud.es (L.G.-Á.); cgarciag@riojasalud.es (C.G.-G.); 2 Servicio de Microbiología Clínica y Enfermedades Infecciosas, Hospital General Universitario Gregorio Marañón, 28007 Madrid, Spain; 3 Instituto de Investigación Sanitaria Gregorio Marañón, 28007 Madrid, Spain; 4 CIBER Enfermedades Respiratorias-CIBERES (CB06/06/0058), 28029 Madrid, Spain; 5 Facultad de Medicina, Universidad Complutense de Madrid, 28040 Madrid, Spain; 6 Servicio de Enfermedades Infecciosas, Hospital Universitario Marqués de Valdecilla IDIVAL, 39008 Santander, Spain; mcarmen.farinas@scsalud.es (M.d.C.F.-Á); manuel.gutierrezc@scsalud.es (M.G.C.); 7 CIBER de Enfermedades Infecciosas-CIBERINFEC (CB21/13/00068), Instituto de Salud Carlos III, 28029 Madrid, Spain; nufernan@gmail.com; 8 Facultad de Medicina, Universidad de Cantabria, 39011 Santander, Spain; 9 Servei de Malalties Infeccioses, Hospital Universitari Vall d’Hebron, 08035 Barcelona, Spain; 10 Departamento de Medicina Universitat Autònoma de Barcelona, 08193 Barcelona, Spain; 11 Servicio de Medicina Interna-Infecciosas, IMIB, Hospital Clínico Universitario Virgen de la Arrixaca, 30120 Murcia, Spain; elisagv@um.es (E.G.-V.); emoralescudero@hotmail.com (E.M.-E.); 12 Facultad de Medicina, Universidad de Murcia, 30100 Murcia, Spain; 13 Servicio de Enfermedades Infecciosas, Hospital Universitario de Canarias, 38320 Tenerife, Spain; mmsocas@hotmail.com (M.d.M.A.-S.); dacilrosado@hotmail.com (D.G.-R.); 14 Unidad de Enfermedades Infecciosas, Hospital Universitario Virgen de las Nieves, 18014 Granada, Spain; chidalgo72@gmail.com; 15 Servicio de Cardiología, Hospital Universitario Puerta de Hierro-Majadahonda, 28222 Madrid, Spain; fdominguezrodriguez@gmail.com; 16 Servicio de Enfermedades Infecciosas, Hospital Universitario de Cruces, 48903 Bilbao, Spain; anejosune.goikoetxeaagirre@osakidetza.eus; 17 Servicio de Medicina Interna, Hospital Universitario de Álava, 01009 Vitoria, Spain; juancarlos.gainzarainarana@osakidetza.eus; 18 Unidad de Cuidados Intensivos Cardiológicos, Hospital Universitario Central de Asturias, 33011 Oviedo, Spain; angelesresteban@gmail.com; 19 Unidad de Enfermedades Infecciosas, Hospital General de Gran Canaria Dr. Negrín, 35010 Las Palmas de Gran Canaria, Spain; xerach.bosch@gmail.com

**Keywords:** infective endocarditis, blood culture negative endocarditis, *Bartonella* endocarditis, *Bartonella* spp.

## Abstract

Blood culture negative endocarditis (BCNE) is frequent in infective endocarditis (IE). One of the causes of BCNE is fastidious microorganisms, such as *Bartonella* spp. The aim of this study was to describe the epidemiologic, clinical characteristics, management and outcomes of patients with *Bartonella* IE from the “Spanish Collaboration on Endocarditis-Grupo de Apoyo al Manejo de la Endocarditis infecciosa en España (GAMES)”cohort. Here we presented 21 cases of *Bartonella* IE. This represents 0.3% of a total of 5590 cases and 2% of the BCNE from the GAMES cohort. 62% were due to *Bartonella henselae* and 38% to *Bartonella quintana*. Cardiac failure was the main presenting form (61.5% in *B. hensalae*, 87.5% in *B. quintana* IE) and the aortic valve was affected in 85% of the cases (76% in *B. henselae*, 100% in *B. quintana* IE). Typical signs such as fever were recorded in less than 40% of patients. Echocardiography showed vegetations in 92% and 100% of the patients with *B. henselae* and *B. quintana*, respectively. Culture was positive only in one patient and the remaining were diagnosed by serology and PCR. PCR was the most useful tool allowing for diagnosis in 16 patients (100% of the studied valves). Serology, at titers recommended by guidelines, only coincided with PCR in 52.4%. Antimicrobial therapy, in different combinations, was used in all cases. Surgery was performed in 76% of the patients. No in-hospital mortality was observed. One-year mortality was 9.4%. This article remarks the importance for investigating the presence of *Bartonella* infection as causative agent in all BCNE since the diagnosis needs specific microbiological tools and patients could benefit of a specific treatment.

## 1. Introduction

Infective endocarditis (IE) is a fatal condition without treatment with an incidence oscillating worldwide between 1.5 and 9.6 cases/100,000 inhabitants [1] although in developed countries it is estimated between 3.1 and 3.7 episodes per 100,000 inhabitants per year [2,3]. The highest incidence occurs in elderly patients with degenerative heart disease and comorbidities such as chronic liver disease, kidney failure and neoplasms [4,5]. In our environment and according to a prospective multicenter registry of 25 Spanish hospitals that analyzed 1804 patients, it has been observed that it mainly affects men (68%) of 55–77 years age, associated, in a considerable percentage, with health procedures or nosocomial environment [6]. Regarding the etiological agents involved in its etiology, 80% of the cases are due to Gram-positive bacteria, mainly due to *Staphylococcus* spp., followed by *Streptococcus* spp. and a long list of other bacterial species [6,7].

Blood culture remains the most frequently used technique for etiological diagnosis, although other techniques such as molecular biology have been generalized for microbiological diagnosis. In the Spanish series, 14.7% were blood culture negative endocarditis (BCNE) and in 9.1%, despite using other techniques such as molecular and serological, the etiology was unknown [6]. In the large series of the International Collaboration on Endocarditis in which 2781 patients were collected, BCNE accounted for 11.1% [7]. However, depending on the place and the available techniques where the studies were carried out, BCNE can increase up to 68 and 70% [8,9]. This fact is important since the absence of a microbiological diagnosis, at least in our environment, could be an independent predictor of mortality [10]. There are many causes of BCNE, including difficult-to-culture microorganisms among which are *Bartonella* spp.

*Bartonella* spp. are fastidious Gram-negative bacilli, facultative intracellular bacteria, belonging to the Alpha-2 subgroup of the phylum Proteobacteria and includes more than 35 validated species and three subspecies [11,12]. These bacteria are usually transmitted to humans through animal bites or scratches (cats, dogs and other animals) or by scratch inoculation of infected flea and body louse feces [13]. In addition, sand-fly vector-competence was proven for transmission of *Bartonella bacilliformis* in Peru during the last century [14]. Other arthropods such as ticks, head lice, bedbugs, bat flies and mites have been associated with human *Bartonella* spp. infections, but their role as vectors remains to be verified [13,15,16,17].

*Bartonella* spp. have been associated with an expanding clinical spectrum in humans and animals. Some species, such *as Bartonella bacilliformis* (Oroya fever, Verruga Peruana or Carrión’s disease) cause potentially life-threatening illness, but are limited geographically by the transmitting vector (confined to the highlands of Peru, Colombia and Ecuador) whereas other species such as *Bartonella quintana* are transmitted by lice under poor hygienic conditions throughout the world. *B. quintana* the etiological agent of trench fever, also is cause of bacillary angiomatosis and peliosis hepatis in HIV patients, chronic bacteremia, chronic lymphadenopathy, as well as BCNE [13,18]. *Bartonella henselae* is the most frequent etiological agent of a sub-acute and chronic lymphadenopathy named cat-scratch disease (CSD) in children and teenagers and occurs throughout the world. A subset of CSD patients develop severe or systemic disease manifestations, including endocarditis, osteomyelitis, granulomatous hepatitis and hepatosplenic abscess [19,20,21,22,23,24,25,26]. Besides these, at least 15 other *Bartonella* spp. have been associated with human diseases or have been detected in humans.

Regarding endocarditis due to *Bartonella* spp., at least eight species have been involved. *B. henselae* and *B. quintana* are present in more than 99% of the cases, although other *Bartonella* spp. such as *Bartonella elizabethae*, *Bartonella vinsonii subsp. berkhoffii*, *B. vinsonii subsp. arupensis*, *Bartonella koehlerae*, *Bartonella alsatica* and “*Candidatus* Bartonella mayotimonensis”, have been occasionally reported [15,27,28,29,30,31,32,33,34]. In any case, there are few published series of *Bartonella* IE and when searching about this condition, there are some differences in the epidemiology, such as the distribution of the involved *Bartonella* spp., and the clinical course. 

The aim of this study was to describe the epidemiologic, clinical characteristics, management and outcomes in a cohort of patients with IE due to *Bartonella* spp. from Spain. 

## 2. Results

### 2.1. Epidemiological Data

A total of 5590 cases of IE were recorded in the GAMES Cohort between 2008 and 2020. From the total, 832 (14.9%) were diagnosed with BCNE of which, 17 (2%) were IE caused by *Bartonella* spp. Four additional patients from another hospital were included. Therefore, a total of 21 cases were included. Thirteen of them were caused by *B. henselae* (61.9%) and the remaining by *B. quintana* (38.1%). No other species of *Bartonella* were detected. 

The median age of the patients with *Bartonella* endocarditis was 49 years (IQR: 40–59 years), and mean age was 51.8 years (28–88 years). According to the species of *Bartonella*, median age of IE caused by *B. henselae* was 55 years (IQR: 42–58) and 42 years (IQR: 34.5–61.5) with IE caused by *B. quintana*. Thirteen were men (61.9%), seven of them diagnosed with IE caused by *B. henselae* and six by *B. quintana*. Eight of the patients with *B. henselae* IE were from the north of Spain (8/21 (38.1%) of the total; 8/13 (61.5%) with *B. henselae*), three from the Canary Islands (3/21 (14.3%) of the total; 3/13 (23.1%) with *B. henselae*), one from the South of Spain and one from a migrant patient from Morocco. Three patients with *B. quintana* IE were from the South of Spain (3/21 (14.3%) of the total; 3/8 (37.5%) with *B. quintana*), one from the Canary Islands one (1/21 (4.8%) of the total; 1/8 (12.5%) with *B. quintana*), one from Morocco and another from central Spain. The two remaining patients were from Moldavia and from Pakistan. 

Five of the thirteen patients (38.5%) of the *B. henselae* group and three of the eight with *B. quintana* (37.5%) reported cat or cat flea exposure history. History of alcohol abuse was recorded in one patient with *B. henselae* IE (1/13; 7.7%) and in two with IE due to *B. quintana* (2/8; 25%). None of the patients had previous disease caused by *Bartonella* spp., none had human immunodeficiency virus (HIV) infection or were intravenous drug users. Body lice contact or undomiciled/homeless situation were not reported in any patient. 

Main epidemiological, clinical and outcomes characteristics are shown in Table 1 and Table 2.

### 2.2. Clinical Features

According to the medical records, 14 of the 21 patients had previous heart disease (9/13 (69.2%) of the patients with IE due to *B. henselae* and 5/8 (62.5%) of the patients with IE due to *B. quintana).* Ten of these patients (41.6%) had previous valve disease (5/13 (38.4%) of the patients with *B. henselae* IE and 5/8 (62.5%) of the patients with *B. quintana* IE). Prosthetic valve was present only in one of the *B. henselae* patients (1/13; 7.7%) and two of this group also had a coronary disease (2/13; 15.4%), three had congenital heart disease (3/13; 23.1%). Two of the patients with *B. quintana* IE (2/8; 25%) and one of the *B. henselae* group (1/13; 7.7%) presented previous heart failure. Other medical conditions are shown in Table 2.

Cardiac failure was the main presenting form of endocarditis caused by *Bartonella* spp. described in our patients. All but one of the patients with *B. quintana* IE (7/8; 87.5%) presented heart failure and 8/13 (61.5%) of those infected with *B. henselae* (NYHA class IV: 3/8 (37.5%) of patients with IE due to *B. henselae* and 5/7 (71.4%) of the patients with *B. quintana*; NYHA class III: 4/8 (50%) of patients with *B. henselae* IE and 2/7 (28.6%) of the patients with *B. quintana*; NYHA class I just one patient (1/8; 12.5%) with *B. henselae* IE). Fever was recorded in 5/13 (38.5%) of patients infected by *B. henselae* and 3/8 (37.5%) of patients infected by *B. quintana*. 

Physical examination focusing on cardiac auscultation showed a new regurgitant murmur in 4/13 (30.8%) and 5/8 (62.5%) of patients with *B. henselae* and *B. quintana* IE, respectively, while classical clinical signs of endocarditis, such as petechiae, were only observed in one patient of the *B. henselae* group. Embolisms were observed in 4/13 (30.8%) and 1/8 (12.5%) of patients with *B. henselae* and *B. quintana*, respectively, (all of them affecting CNS in the *B. quintana* group while only one affected this structure in the *B. henselae* patients). New renal function impairment was observed in four of the 13 patients infected by *B. henselae* (30.8%) and in 5/8 (62.5%) by *B. quintana*. Two patients had splenomegaly, one of each group (1/13 (7.7%) of *B. henselae* and 1/8 (12.5%) of *B. quintana*). Only one patient with *B. henselae* IE presented glomerulonephritis (1/13; 7.7%). One patient of the *B. quintana* group presented a new conduction abnormality (1/8; 12.5%) and one of the *B. henselae* group had septic shock (1/13; 7.7%).

Echocardiography was performed for all 21 patients: both transthoracic echocardiography (TTE) and transesophageal echocardiography (TOE) for 9/13 (69.2%) of patients with *B. henselae* and for 5/8 (62.5%) of patients *B. quintana;* TTE for 12/13 (92.3%) and 100% patients with *B. henselae* and *B. quintana*, respectively and TOE for 10/13 (76.9%) and 5/8 (62.5%) of patients with *B. henselae and B. quintana*, respectively. Echocardiography showed vegetations in 12/13 (92.3%) and 100% of the patients with *B. henselae* and *B. quintana,* respectively. The size of the vegetations was between 5 and 25 mm in patients infected by *B. henselae* (mean ± SD: 13.8 ± 6.5 mm) and between 7 and 13 mm in those infected by *B. quintana* (mean ± SD: 10.6 ± 2.2 mm). 

The involved valve was the aortic in 18/21 cases (85.7%), although seven of them (7/18; 38.9%) also presented mitral valve involvement. In three patients the unique valve involved was the mitral (3/21; 14.3%). According to the *Bartonella* spp. implicated, in patients with *B. henselae* IE, the aortic valve was affected in ten (10/13; 76.9%), three of them also presented mitral valve involvement. In three patients (3/13; 23.7%) the only valve affected was the mitral. Nevertheless, in patients infected by *B. quintana* the aortic valve was affected in all patients with half of them also presenting mitral valve involvement. 

Native valve was affected in all but one (20/21; 95.2%). Prosthetic valve was affected only in one patient infected by *B. henselae*. Intracardiac complications were observed in 12/21 (57.1%) of the patient (9/13 (69.2%) patients infected by *B. henselae* and in 3/8 (37.5%) infected by *B. quintana*): valve perforation was detected in 3/21 (14.3%) of patients of which, two of them were infected by *B. henselae* (2/13; 15.4%) and one by *B. quintana* (1/8; 12.5%), pseudoaneurysm in 6/21 (28.6%) of the patients, five of them infected by *B. henselae* (5/13; 38.5%) and one by *B. quintana* (1/8; 12.5%), and abscess in four of the *B. henselae* group (4/13; 30.8%). 

Laboratory records showed alterations in C-reactive protein and erythrocyte sedimentation rate values. These data are shown in Table 2. Anemia (hemoglobin (Hb) levels < 12.0 g/dL in women and < 13.0 g/dL in men) was observed in ten of the thirteen patients infected with *B. henselae* (76.9%) and in six of the eight patients infected with *B. quintana* (75%). 

Surgery was performed in 16/21 patients (76.2%). Urgent surgery was performed in three cases (3/16; 18.8%) and emergency surgery in one (1/16; 6.3%). 

### 2.3. Microbiological Diagnosis

Table 3 shows the techniques used for the microbiological diagnosis, the results obtained depending of the applied technique and the coincidence among them. It should be noted that serology was carried out in 17/21 patients (80.9%) and, initially, 6/17 (35.3%) had titers < 800 and 9/17 (53%) had titers ≥ 512. In the five cases in which the diagnosis was only made by serology, either the serologies were positive only for *B. henselae* while negative for *B. quintana*, or there was a greater rise in titer for *B. henselae* (more than three titers). 

### 2.4. Treatment and Outcome

According to European guideliness, the proposed therapy is doxycycline 100 mg/12 h orally for four weeks plus gentamicin (3 mg/24 h) i.v. for two weeks. However, several therapeutic regimens have been reported, including aminopenicillins (ampicillin or amoxicillin, 12 g/24 h i.v.) or cephalosporins (ceftriaxone, 2 g/24 h i.v.) combined with aminoglycosides (gentamicin or netilmicin) [35]. Antimicrobial therapy for each patient is shown in Table 4. 

Median days of antibiotic treatment was 49 (range: 42–90). Median days of hospital admission was 41 (IQR: 29–54). Two patients were readmitted (2/21; 9.5%). Neurological or cardiac sequelae (e.g. dysarthria, hemiparesis, hemiplegia, heart failure) were present in three patients (3/21; 14.3%). None of the patients had a relapse. None of the patients died during the hospital stay. Two patients died two and three months after the process in relationship with the IE. 

## 3. Discussion

To date, IE remains a serious disease all over the world, including developed countries. In most cases, Gram-positive cocci are the causative agents but fastidious organisms such as *Bartonella* spp., *Coxiella burnetii*, *Tropheryma whipplei* and others must be considered. In the GAMES cohort, *Bartonella* spp. is the third most common cause of BCNE behind *C. burnetii* and *T. whipplei* [36]. There a few *Bartonella* series and here we present a new one that is of the most numerous series from a developed country. 

The prevalence of *Bartonella* endocarditis varies among countries. A majority of cases of *Bartonella* IE have been reported from Europe and the Americas. However, cases from Asia, Africa, and Australia have been also documented, suggesting a worldwide distribution [37,38,39,40,41,42,43,44,45,46,47]. In Europe, *Bartonella* spp. account for 0–4.5% of the infective endocarditis and 20–30% of BCNE endocarditis [48,49,50,51,52]. In UK *Bartonella* IE represent 1.1% of IE [53]. However, *Bartonella* spp. as a causative agent of infective endocarditis reaches 9.8% in Tunisia or even 15.6% in Algeria [39,54]. In Spain, last published data of the GAMES cohort, which included patients recruited beteween 2008 and 2012, estimated that *Bartonella* spp. were the cause of endocarditis in 0.2% of the cases [6,36]. Here we report the main characteristics of 21 patients affected by *Bartonella* spp. Taking into account that just 17 of them were from hospitals belonging to the GAMES Cohort, the rate of *Bartonella* spp. as a causative agent of endocardits recorded in the GAMES Cohort between 2008 and 2020, is 0.3% of the total of IE, and 2% of the BCNE registered. These data are far below those reported in the previously cited series. Nevertheless, the prevalence of *Bartonella* endocarditis could be underestimated due to the difficulties involved in its identification. Probably, data here do not represent the true incidence of *Bartonella* IE in our country but in our opinion is the best approximation. 

To diagnose endocarditis due to *Bartonella* spp. is not easy since the clinical presentation is not typical. Fever is not presesent in more than half of the cases and patients usually come to the hospital due to complications such as cardiac failure. This fact is very important since it reinforces the need to request echocardiography in these patients, that is the gold standard for diagnosing endocarditis. Other typical signs such as embolic events are also rare in these patients. However, as in other IE, the existence of previous valve disease is a risk factor for endocarditis due to *Bartonella* spp. [19]. Preexisting valvular abnormalities are a common condition in patients with *Bartonella* endocarditis, in more than 40% of the cases, reaching 62.5% of the patients with *B. quintana* IE. As previously described, results presented here show that the aortic valve is the most affected one, affecting all cases of *B. quintana* here described and almost 80% of the endocarditis due to *B. henselae* [19,24,39,43,47,48,52,53,54,55,56,57].

The epidemiology in the cases presented here differs from most series. According to the literature, *B. quintana* is the causative agent in approximately 75% of all cases, while *B. henselae* occurs in around 25% of all infective endocarditis cases [47]. However, it is important to note that in the data presented here, *B. henselae* accounts for almost 62% of the cases while the remaining are due to *B. quintana. B. henselae* as the main *Bartonella* spp. causing IE has been only reported in just two other series [43,55].

None of our patients referred body lice contact or an undomiciled/homeless situation and alcohol abuse was observed only in very few cases. These factors, which have been classically associated with *B. quintana* infection [57], could explain the low prevalence of *B. quintana* endocarditis in our environment campared with other series such as the French, Tunisian or Algerian ones where the prevalence of *B. quintana* IE is high [19,39,54]. It should also be noted that, in our series, a not insignificant percentage of patients with *B. quintana* IE were immigrants. These patients were from Moldavia, Pakistan and Morocco but although they were diagnosed in Barcelona, Spain, the infection most likely happened in their country of origin since two of them had just arrived in Barcelona and, since in the case of the Moroccans, they spent long periods in Morocco. Other classical epidemiological factors such as cat contact, which has been associated with an increased risk of *B. henselae* infection, is not a prominent factor in our series. Moreover, both species of *Bartonella* described here affect men more frequently than women and, the age of presentation (49 years) is lower than in the endocarditis caused by the “usual agents“ [6,7] and even lower than in patients with *Bartonella* IE recorded in the French series [19]. 

The systematic use of serological and molecular assays in the study of BCNE has improved the diagnosis of this entitity. When *Bartonella* IE is suspected, it can be diagnosed by blood or valve culture (not very sensitive with the usual techniques), molecular assays such as PCR on blood or excised tissue, that in our series is the most usuful technique, and by serological procedures. Other techniques such as silver nitrate-based staining (Warthin-Starry stain) in affected tissues are also very suggestive [58]. Before the incorparation of molecular techniques, most of the cases of *Bartonella* endocarditis were diagnosed using serological tests, especially immunofluorescence assay (IFA) that to date are recommended in the guideliness and by experts [35,59]. In the series presented here, it is remarkable that serology has a poor correlation for the diagnosis if the cut-off point recommended in the guideliness is used [35,59]. As previously described [19], we have also observed that 35% of the patients with definitive endocarditis produced by *Bartonella* spp. confirmed by PCR do not have the recommend cut-off point of IgG ≥ 800 by IFA. In this sense, molecular procedures are likely to become a key tool to improve BCNE diagnostics and contribute to a better understanding of the aetiology [60]. Moreover, the limitations of serological tests should be recognized due to the high level of serological cross-reactions between *Bartonella* and *Chlamydia/Chlamydophila* [61] and the difficulty to distinguish between species of *Bartonella* which further complicates the diagnosis of this type of endocarditis. In the last few years, new methods improving the sensitivity to diagnose *Bartonella* infections have been described that may be validated for the use in clinical practice, e.g., a method based on a liquid enrichment *Bartonella* alphaproteobacteria growth medium (BAPGM) followed by PCRs for the amplification of *Bartonella* spp. [62].

Most of the patients in this series, although in different combinations, had antimicrobial therapy according to guideliness. It is important to emphasize that most of the patients presented here required surgical treatment, perhaps since the diagnosis of *Bartonella* endocarditis, and in general of BCNE, was later and optimal treatment was not always included early. Mortality due to *Bartonella* IE in the first descriptions was significant with rates ranging 7 to 30%. However, in recent times mortality rates closer to 7% have been shown, probably due to the improved diagnostic and therapeutic measures [9,47,51,63,64,65,66,67,68]. These data are in agreement with those reported here (mortality rate observed here was 9.4%).

## 4. Materials and Methods

### 4.1. Patients’ Recruitment

Patients diagnosed with *Bartonella* IE were recruited from a registry of the “Spanish Collaboration on Endocarditis - Grupo de Apoyo al Manejo de la Endocarditis infecciosa en España (GAMES)”. GAMES is formed by 25 Spanish hospitals that makes a prospective registry including aspects related to IE. Furthermore, four cases from the Vall d´Hebron Hospital (Barcelona), which is not included in the Games Cohort, were also recruited. The necessary data for this study of patients from Vall d´Hebron Hospital were obtain retrospectively from their medical records by their physician. Between 1 January 2008 and 31 December 2020, multidisciplinary teams prospectively completed a standardized case report form containing epidemiological, clinical, biological (including main hematological and biochemical values) and therapeutic data for each patient. This case report form was designed to record any type of endocarditis and does not include specific variables for *Bartonella* endocarditis, therefore, some data of interest for this type of endocarditis, such as contact with cats, fleas, body lice, undomiciled/ homeless situation or history of alcohol abuse (considering alcohol abuse, according to the WHO, the consumption of more than 40 g for men and more than 25 g for women), were retrospectively reviewed from the clinical record of each patient. Due to this information was restricted to what the clinician has entered into the medical records some data could be underestimated. Regional and local ethics committees approved the study (Comité Ético para la Investigación Clínica-Regional de la Consejería de Sanidad de la Comunidad de Madrid, code: 18/07; Date: 11 January 2008), and patients gave their informed consent for entering the cohort [6].

### 4.2. Definitions

IE and *Bartonella* infection as causative agent was defined according to the modified Duke criteria [69]. In our clinical practice a routine protocol that includes serology to *Coxiella burnetii*, *Bartonella* spp., *Legionella* spp. and *Brucella* spp. was completed in all patients with BCNE. When valve surgery was carried out molecular assays (PCR) or culture were performed. Molecular tests were performed using *Bartonella* spp. *rpoB* gene and broad-range 16S rRNA gene and subsequent sequencing [70,71]. Serological studies were carried out in each clinical laboratory included in the study using commercial immunofluorescence assays. Taking into account the possibility of cross-reactions between *Bartonella* spp., the species was determined based on serological assays if results were positive only for one species or if there was at least a three-fold difference in titer against one species compared to the other one. 

### 4.3. Statistics

Statistical analysis was performed using SPSS, version 26.0 (IBM Corporation, Armonk, NY, USA).

## 5. Conclusions

*Bartonella* IE is not a frequent cause of IE that should be suspected in all patients with BCNE. In these cases, the index of suspicion should be high even if contact with animals has not been reported or there are no risk factors such as body lice parasitization. A serology for *Bartonella* spp. negative or lower than the recommended cut-off does not exclude the possibility of a *Bartonella* IE. The prognosis of *Bartonella* IE was better in this study than in IE due to other causes observed in the Games Cohort [6,10], and involves valve replacement in a high percentage of the cases.

## Figures and Tables

**Table 1:** Main epidemiological, clinical, and outcome characteristics of the patients with *Bartonella* spp. Endocarditis.

	*Bartonella henselae* IE	*Bartonella quintana* IE	Total
Nº patients	13	8	21
Age median (IQR)	55 (42–58)	42 (34.5–61.5)	49 (40–59)
Male	7 (53.8%)	6 (75%)	13 (61.9%)
Previous heart disease	69.2%	62.5%	66.7%
Alcohol abuse	7.7%	25%	14.3%
Cats/Fleas/Body lice contact	38.5%	37.5%	38%
Median age-adjusted Charlson comorbidity index (IQR)	2 (1–4)	0 (0–2.5)	1 (0.5–4.5)
Affected valve/s Aortic/Mitroaortic	76.9%/23.7%	100%/50%	85.7%/38.9%
Cardiac failure	61.5%	87.5%	66.7%
Fever	38.5%	37.5%	42.8%
Mean vegetations size (mm) ±SD	13.8 ± 6.5	10.6 ± 2.2	12.7 ± 5.6
Intracardiac complications	69.2%	37.5%	57.1%
Surgery	61.5%	100%	76.2%
Anemia	76.9%	75%	76.2%
Median duration of antimicrobial therapy (IQR)	46 (42–92)	70.5 (42–110)	49 (42–103)
Days of hospital admission median (IQR)	40 (28–52.5)	43 (38.5–55.5)	41 (28.5–54.5)
Sequelae	15.4%	12.5%	14.3%
Relapse	0	0	0
Death (1st year)	2 (15.4%)	0	2 (9.5%)

IE, infective endocarditis; IQR, interquartile range.

**Table 2:** Epidemiological, clinical, and outcome characteristics of the 21 patients.

Patient Id.	1	2	3	4	5	6	7	8	9	10	11	12	13	14	15	16	17	18	19	20	21
*Bartonella* spp.	BH	BH	BH	BH	BH	BH	BH	BH	BH	BH	BH	BH	BH	BQ	BQ	BQ	BQ	BQ	BQ	BQ *	BQ
Age	42	58	44	40	82	88	59	47	57	57	40	55	40	33	35	72	28	66	60	49	35
Sex	F	M	M	F	F	F	M	F	M	M	M	F	M	F	M	M	M	M	M	F	M
Country of residence	Spain	Spain	Spain	Spain	Spain	Spain	Spain	Spain	Spain	Spain	Spain	Spain	Morocco	Spain	Spain	Spain	Spain	Spain	Moldova	Morocco	Pakistan
Year of diagnosis	2014	2017	2010	2012	2018	2010	2013	2019	2019	2018	2020	2021	2014	2012	2016	2013	2019	2009	2011	2018	2016
Malignancies	N	N	N	N	N	N	N	N	N	N	N	N	N	N	N	N	N	N	N	N	N
Previous heart disease	Y	N	N	Y	Y	N	Y	Y	N	Y	Y	Y	Y	Y	N	Y	N	Y	N	Y	Y
Alcohol abuse	N	N	Y	N	N	N	N	N	N	N	N	N	N	Y	Y	N	N	N	N	N	N
Other historical conditions	CTD, IT	CLD, HT, HL, VD	RFI, HD, PU	HT, IT, CTD, RFI	CLD, DM, HT, CD, HL	HT, CVD	N	N	CD	DM, HT, HL	N	CTD	HT, CVD, TA.	N	N	DM, HT, HL, VD, RFI.	N	HT, HL, RFI	HL	N	N
Cats/Fleas/Body lice contact	Y	Y	N	N	Y	N	N	Y	N	N	N	Y	ND	Y	N	N	Y	N	Y	ND	ND
Age-adjusted charlson comorbidity index	1	3	5	4	7	5	1	0	1	2	1	2	1	1	0	7	0	9	2	0	0
ETE/ETT	BOTH	BOTH	BOTH	BOTH	TOE	TTE	BOTH	TTE	BOTH	BOTH	TTE	BOTH	BOTH	TTE	BOTH	BOTH	TTE	BOTH	BOTH	BOTH	TTE
Affected valve/s	AO	AO	AO-MIT	AO	MIT	AO-MIT	MIT	AO	MIT	AO	AO-MIT	AO	AO	AO-MIT	AO-MIT	AO	AO	AO	AO-MIT	AO-MIT	AO
Vegetations size (mm)	9	12	5	NV	8	15	25	25	ND	12	ND	10	17	ND	ND	12	ND	13	7	12	9
Cardiac failure (NHYA)	N	Y(III)	N	Y(III)	N	Y(I)	N	Y(III)	Y(III)	Y(IV)	Y(IV)	N	Y(IV)	NO	Y(III)	Y(IV)	Y(III)	Y(IV)	Y(IV)	Y(IV)	Y(IV)
Fever	N	N	Y	Y	Y	Y	Y	N	N	N	N	N	Y	N	N	Y	N	N	Y	Y	N
Intracardiac complications	N	N	Y	Y	Y	Y	N	Y	Y	Y	N	Y	Y	N	Y	N	N	N	Y	Y	N
Embolisms	Y	N	N	N	N	Y	N	N	Y	Y	N	N	N	N	Y	N	N	N	N	N	N
Surgery	Y	Y	Y	Y	N	N	N	Y	Y	N	N	Y	Y	Y	Y	Y	Y	Y	Y	Y	Y
CRP (mg/L) **	1.5	57.5	5.6	108	3	1.4	20.9	ND	11	51	70	11	ND	ND	181	ND	4.4	49	ND	ND	7.8
ESR (mm/h)	44	141	ND	32	13	ND	ND	ND	25	ND	ND	ND	120	ND	ND	ND	120	123	118	ND	ND
AST/ALT (U/L)	115/208	17/12	19/8	30/26	23/16	UNK	102/107	20/19	32/61	34/30	33/25	18/8	34/30	15/16	40/35	2387/2224	34/19	68/10	17/16	10/5	14/11
Microbiological diagnosis	SER/ PCR	SER/ PCR	SER	SER/ PCR	SER	SER	SER	PCR	SER	SER/ PCR	SER/ PCR	SER/ PCR	PCR	PCR	SER/ PCR	PCR	PCR	SER/ PCR	SER/ PCR	SER/ PCR	SER/ PCR
Antimicrobial Duration (days)	207	42	35	35	116	46	42	58	42	68	42	46	123	49	198	25	35	51	90	90	130
Days of hospital admission	40	28	3	47	95	47	51	34	54	28	28	23	57	38	56	55	29	56	39	45	41
Sequelae	N	N	N	N	Y	N	N	N	Y	N	N	N	N	N	Y	N	N	N	N	N	N
Outcome	A	A	A	D (IE RELATED)	D (IE RELATED)	D (IE NOT RELATED)	A	A	A	A	A	A	A	A	A	A	A	A	A	A	A

BH, *Bartonella henselae*; BQ, *Bartonella quintana*; F, female; M, male; N, no; Y, yes; CTD, connective tissue disease; CLD, chronic lung disease; HT, hypertension; HL, hyperlipidemia; VD, vascular disease; RFI, renal function impairment; HD, hepatic disease; PU, peptic ulcer; IT, immunosuppressive therapy; DM, diabetes mellitus; CD, coronary disease; CVD, cerebrovascular disease; TA, tuberculous adenitis; ND, not documented; TOE, transesophageal echocardiography; TTE, transthoracic echocardiography; AO, aortic valve; MIT, mitral valve; NV, not vegetation; NHYA, New York Heart Association; CRP, C-reactive protein; ESR, erythrocyte sedimentation rate; AST, aspartate aminotransferase; ALT, alanine aminotransferase; SER, serology; PCR, polymerase chain reaction A, alive; D, death. * SAMS coinfection; ** Highest value during the episode.

**Table 3:** Microbiological diagnosis.

Microbiological Technique	N (%)	*B. henselae*	*B. quintana*
**Blood culture**	1	0	1
**PCR** Blood Valve/vegetation Blood and valve	16 (76.2) 2 (9.5) 16 (76.2) 2 (9.5)	8 1 8 1	8 1 8 1
**Serology ^a^**	17 (80.9)	11	5
**More than one technique**	12 (57.1)	6	6
Only culture Only PCR Only serology Culture and PCR Culture and serology PCR and serology Culture, PCR and serology	- 3 (14.2) 5 (23.8) 1 (4.8) - 11 (52.4) -	- 2 5 - - 6 -	- 1 - 1 - 5 -
**Coincidences**	11 (52.4)	6	5

PCR: Polymerase chain reaction (PCR). ^a^ Anti-*Bartonella* spp. IgG ≥ 1/800 (IFA).

**Table 4:** Antimicrobial regimens used.

Patient Id.	Antimicrobials
1	Gentamicin + Ceftriaxone/Gentamicin + Doxycycline
2	Ampicillin + Cloxacillin + Gentamicin/Gentamicin + Doxycycline
3	Ciprofloxacin/Vancomycin + Gentamicin
4	Gentamicin + Doxycycline
5	Gentamicin + Daptomycin + Doxycycline/Daptomycin + Ceftriaxone
6	Gentamicin + Cloxacillin + Ampicillin/Ceftriaxone
7	Ampicillin + Gentamicin + Cloxacillin/Ampicillin + Cloxacillin + Ceftriaxone
8	Gentamicin + Daptmycine + Ceftriaxone/Doxycycline
9	Ampicillin + Cloxacillin + Ceftriaxone/Doxycycline + Imipenem/Gentamicin + Doxycycline + Rifampicin
10	Ceftriaxone + Gentamicin/Doxycycline
11	Ceftriaxone + Daptomycine + Cloxacillin/Doxycycline + Rifampicin
12	Ampicillin + Ceftriaxone + Cloxacillin/Ceftriaxone + Gentamicin + Doxycycline
13	Ampicillin + Ceftriaxone/ Doxycycline
14	Ceftriaxone + Doxycycline + Gentamicin
15	Ceftriaxone + Gentamicin/Cloxacillin + Ampicillin/Rifampicin + Doxycycline + Gentamicin
16	Ampicillin + Cloxacillin + Gentamicine/Daptomycin
17	Ceftriaxone + Ampicillin + Cloxacillin/Gentamicin + Doxycycline
18	Ampicillin + Gentamicin/Daptomycin + Ceftriaxone + Levofloxacin/Gentamicin + Rifampicin + Doxycycline
19	Ceftriaxone/Penicillin + Gentamicin/Doxycycline
20	Doxycycline
21	Ceftriaxone/Doxycycline

## Data Availability

Not applicable.

## Supplementary Materials

The following supporting information can be downloaded at: https://www.mdpi.com/article/10.3390/pathogens11050561/s1, Investigators of Spanish Collaboration on Endocarditis—Grupo de Apoyo al Manejo de la Endocarditis infecciosa en España (GAMES).
